# What is the Optimal Treatment Strategy after Sarcoma R2 Surgery?

**DOI:** 10.1007/s11864-024-01218-z

**Published:** 2024-05-29

**Authors:** Paulina Chmiel, Piotr Rutkowski, Mateusz Spałek, Anna Szumera-Ciećkiewicz, Anna M. Czarnecka

**Affiliations:** 1 Department of Soft Tissue/Bone Sarcoma and Melanoma, Maria Sklodowska-Curie National Research Institute of Oncology, 02-781 Warsaw, Poland; 2 Faculty of Medicine, Medical University of Warsaw, 02-091 Warsaw, Poland; 3 Department of Radiotherapy I, Maria Sklodowska-Curie National Research Institute of Oncology, 02-718 Warsaw, Poland; 4 Department of Pathology, Maria Sklodowska-Curie National Research Institute of Oncology, 02-781 Warsaw, Poland; 5 Biobank Maria Sklodowska-Curie National Research Institute of Oncology, 02-781 Warsaw, Poland; 6 Department of Experimental Pharmacology, Mossakowski Medical Research Institute Polish Academy of Sciences, 02-106 Warsaw, Poland

**Keywords:** Sarcoma, Surgery, Adjuvant therapy, Radiotherapy

## Abstract

Soft tissue sarcomas (STS) are rare tumours of mesenchymal origin, most commonly occurring in the extremity but also in the retroperitoneum. The curative treatment for STS is radical surgery with wide margins, in some cases in combination with perioperative radiotherapy and chemotherapy. Nonradical resection (R2) of STS has been an emerging issue in recent decades, as optimal subsequent management remains debatable. Similarly, there is still no consensus on optimal surgical margins. Combining multiple treatment modalities in adjuvant therapy can achieve local and distant control in patients following surgery with positive margins. Patients who have undergone nonradical resection therefore require additional surgical interventions, and adjuvant radiotherapy resulting in a better prognosis but a higher number of complications. Following non-radical treatment, patients with limb and trunk wall sarcomas and retroperitoneal sarcomas should also undergo increased oncological surveillance. Given the potential issues that may emerge in such clinical situations, it is crucial to up-date the current guidelines to enhance the long-term prognosis of these patients.

## Introduction

Soft tissue sarcomas (STS) are a rare and diverse group of malignancies that account for approximately 1% of all adult cancers [1]. In Europe, the estimated incidence is 4-5 cases per 100,000 individuals per year [2]. The WHO classification identifies over 100 STS subtypes with different prognoses and clinical presentations [3]. STSs can arise anywhere in the body but are most commonly found in the extremities (upper extremities in approximately 12% and lower extremities in approximately 28%) and in the retroperitoneum in 16% of cases [4, 5]. The subtypes that occur most frequently are liposarcomas (20%-25%), leiomyosarcomas (14%), and undifferentiated pleomorphic sarcomas (UPS) (14%) [1]. Personalized treatment for patients with sarcoma is based on the histological type and location of the tumour. Multidisciplinary tumour boards (MDTB) are recommended for the treatment of STSs due to their rarity [6]. These boards should involve surgeons, medical oncologists, radiation specialists, pathologists, and radiologists in high-volume reference centres. The primary curative treatment of STSs is optimal surgical excision with negative margins (no tumour at the margin, R0) [7]. However, the surgical approach can be challenging for several reasons. Firstly, the disease may have extended and infiltrated crucial structures such as vessels, nerves, and organs, making radical resection questionable [8•]. Second, inadequate estimation of the disease may result in a "whoops operation", where surgery is performed assuming the mass is benign, but postoperative pathological examination reveals a diagnosis of sarcoma [9–11]. All non-radical resections for cancer patients vary depending on the type of tumour, with a range of 4.2-53% for sarcomas [12–15]. However, there has been a decrease in these values compared to previous decades, indicating improvements in STS diagnosis and management [16•]. The main issues arising from these situations appear to be their impact on the patient's prognosis, the overall risk of survival deterioration, the likelihood of local recurrence and the presence of distant metastases [17]. The critical role of achieving negative surgical margins to optimize local management of STS is widely recognized. Positive surgical margins predict local recurrence (LR) following STS excision [18, 19]. Although not universally, numerous studies have also linked LR with a reduction in overall survival (OS) [20, 21]. Gross non-radical resection correlates with the aggressive nature of the tumour [22]. Therefore, the failure to achieve this local control often requires more interventions, which are associated with increased morbidity and increased rates of amputation [23]. As mentioned previously, due to the rarity of STS, there are currently no established guidelines for this approach, including how to define optimal negative margins or proceed if negative margins are not possible or are not achieved during surgery [24]. As for now, management depends on the experience of the clinical centre [25]. This review aims to gather information on the epidemiology, prognosis, and treatment of patients after macroscopic nonradical resection of soft tissue sarcomas.

## Definitions

**Fig. 1:**
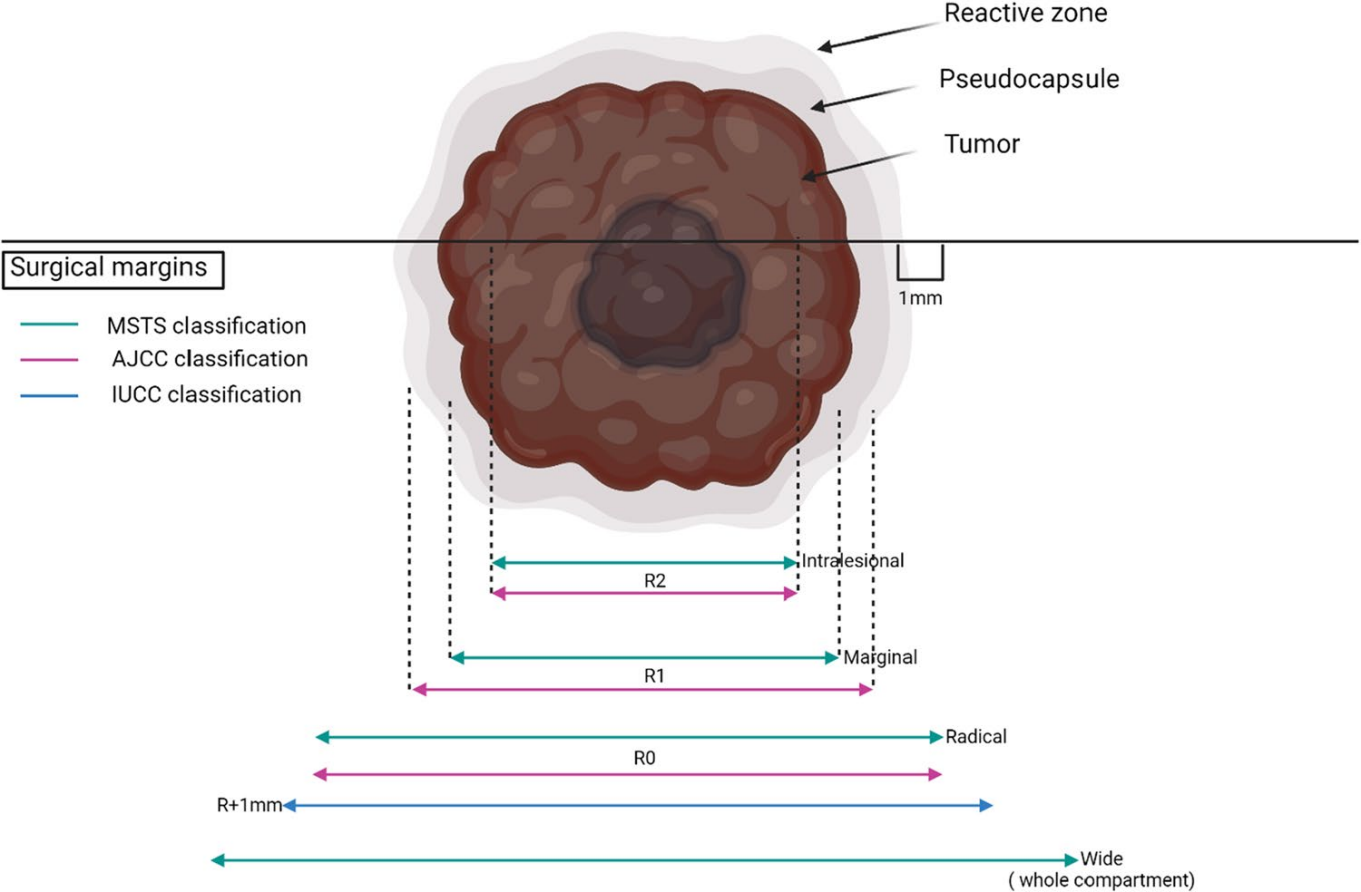
Outlook on surgical margins in treating soft tissue sarcoma according to main classification systems. AJCC- American Joint Committee on Cancer, MSTS- Musculoskeletal Tumour Society, UICC- Union Against Cancer.

**Fig. 2:**
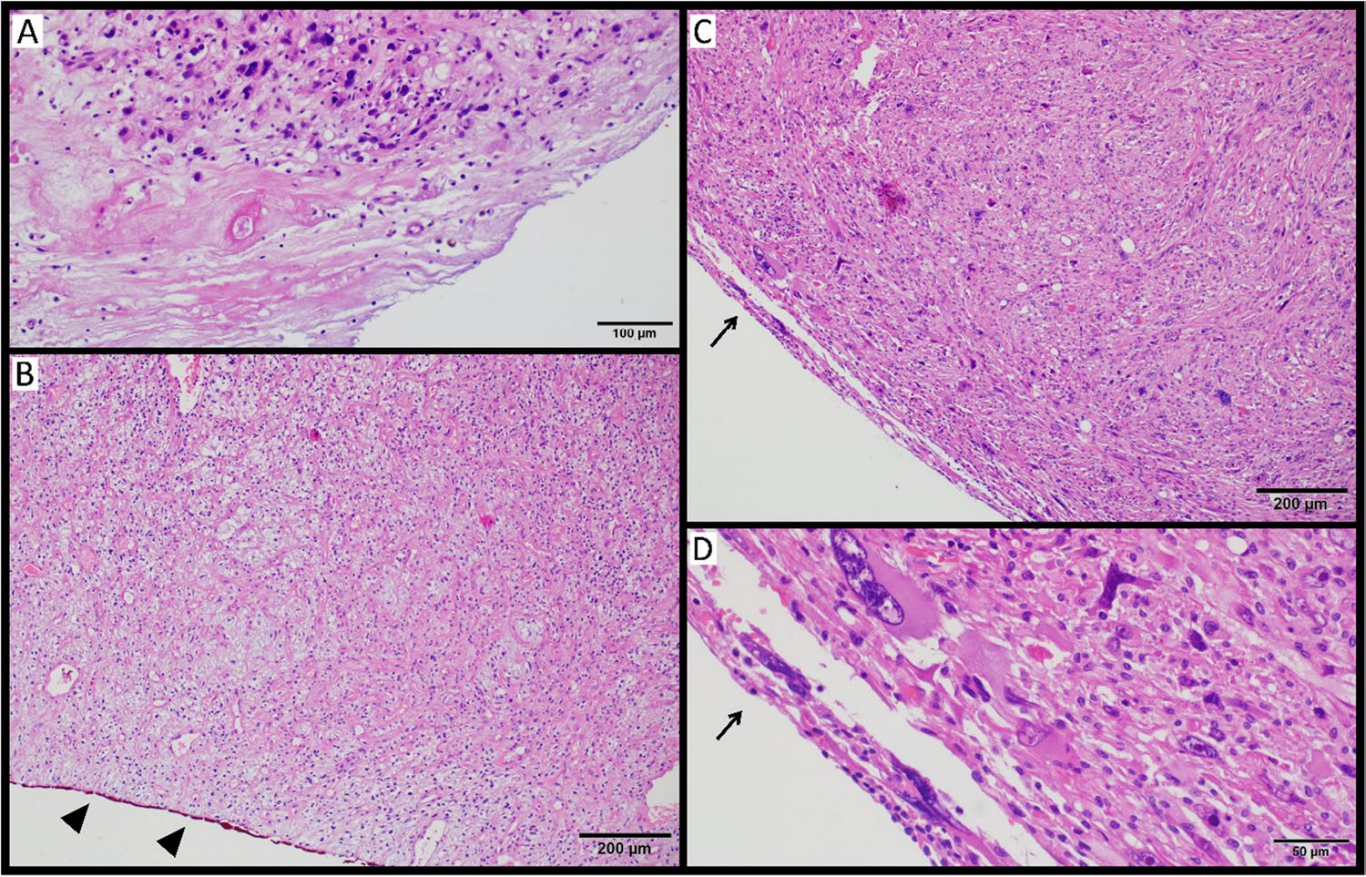
Histopathological view of R2 margins; (**A**) high-grade myxoid liposarcoma partially with necrosis (HE, 200x); (**B**) myxofibrosarcoma with ink-marking margins (arrowheads) during the macroscopic evaluation performed by the pathologist (HE, 100x); (**C**) undifferentiated pleomorphic sarcoma (HE, 100x) with (**D**) highly atypical neoplastic cells at the margins (HE, 400x, arrows).

Several systems have been developed over the years to define surgical margins, particularly in surgical oncology (Figure 1). However, sarcoma guidelines do not provide a specific recommendation for the width of the margin or a standard definition of a negative margin [6, 7]. Enneking created the first classification adopted by the Musculoskeletal Tumour Society (MSTS). This classification established four types of surgical margin: radical (all healthy tissue of the anatomic compartments involved excised en *bloc*), wide (histologically nonreactive normal tissue at the margin), marginal (pseudocapsule present at the margin), and intralesional (tumour present at the margin) [26]. Progress in the treatment strategies of sarcoma patients has led to increased limb-sparing surgeries, necessitating an improvement in the classification mentioned above [27]. The American Joint Committee on Cancer (AJCC) scale is one of the most widely used scales for reporting surgical margins. The system is based on three levels of radicality of resection - R0 (tumour does not reach the resection margins), R1 (tumour cells present in the inked margins of the resected tissue or resection alongside the pseudocapsule), and R2 (macroscopic gross resection) (Figure 2) [28]. There is also a classification that includes the width of the margins, the so-called R + 1 mm classification, developed by the Union Against Cancer (UICC), that changed the concept of the R0 margin by adding the requirement of 1 mm of normal tissue between the tumour and the margin to define a negative margin [29]. The International Soft Tissue Sarcoma Consortium (INSTRuCT) has proposed a more precise definition of surgical margins in the pediatric population. This classification is based on the IUCC proposal, but for the R2 margins, an interesting subdivision has been proposed that more accurately reflects the surgical situation. The R2A category reflects the situation in which the residual tumour can be further resected, R2B when radical surgery cannot be performed without mutilating the patient, and R2C when resection has not been performed due to the advanced stage [30]. The Toronto Margin Context Classification (TMCC) introduced a scale based on the clinical situation. The classification uses a four-tiered scale of surgical margins: negative margins, unplanned positive margins, a planned close but ultimately positive microscopic margin along a critical structure, and a positive margin after re-excision of the tumour bed in patients initially treated with primarily inadequate surgery [31].

## Incidence

Nonradical resection of STSs can result from inadequate diagnosis and treatment in an inexperienced unit, leading to unplanned resection, or from the advancement of the local tumour, making complete resection without a mutilating procedure unfeasible [14]. Occurrence rates of R2 surgery in STSs vary depending on the clinical centre, and unplanned resections are the main reason for unclear margins in sarcoma patients [32]. A large study conducted by NETSARC, which included 26 reference centres for sarcoma treatment, demonstrated that for primary tumour resection, R2 margins were 8.5% in non-NETSARC centres, while patients operated within the network achieved R2 margins in only 4.2% of cases [14]. Furthermore, an updated analysis of this database showed a decrease in R2 resection in recent years from 10.9% to 7.9% [16•]. Smaller retrospective analyses have also confirmed these observations. For example, Melis et al. found a significant difference in the occurrence of non-radical macroscopically procedures, with rates of 6.4% compared to 2.0% (p < 0.01) [33]. Inexperienced practitioners may have an R2 rate of up to 13.5% [15]. However, after inappropriate surgery, imaging studies indicated the presence of residual disease in 53.1% of patients [19]. It is important to note that this study did not provide information on the radicality of the primary surgery. The study found that only half of the cases followed the recommended treatment, resulting in resection of R2 in approximately 25% of the patients [34]. An inaccurate diagnostic process mainly causes these accidental resections, resulting in a macroscopically positive margin. Studies showed that unplanned, nonradical resections are more likely to occur in cases where patients are younger, malignancy is not suspected, tumours are superficially located with a diameter less than 5 cm, and located in the limbs [33, 35–40]. Furthermore, histological subtypes such as malignant fibrous histiocytoma (MFH), malignant peripheral nerve sheath tumour (MPNST), synovial sarcoma, undifferentiated pleomorphic sarcoma (UPS), dermatofibrosarcoma protuberans (DFSP) are more likely to be operated non-radically [15, 36, 39, 41]. The data on liposarcoma are contradictory, as some studies had the lowest rate of nonradical resection in this subtype, and others had the highest rate among the evaluated cohorts [15, 36]. An analysis, independent of surgical inadequacy, showed that factors related to surgery and tumour influenced the positive margins that differentiate the two groups. In the group of resected sarcomas with a positive margin, factors related to the procedure were tumour size, the deep margin was usually positive and tumours were resected from the muscles, UPS and MFH. Among the tumour-related factors that influence the positive margin, resections have been shown to be more likely to have many positive margins, and myxofibrosarcoma was also among the most common subtypes [42]. Radical resection may also be impossible in selected cases without significantly compromising the patient's quality of life. In a location such as the heart, where critical structures are often impossible to preserve, attempts at radical resection of sarcomas result in R2 resection in up to 58% of cases [43]. Retroperitoneal sarcomas (RPS) poses a surgical challenge as margin assessment is difficult. Unlike extremity sarcomas, where margins are more easily defined, in RPS, margins often include crucial internal organs, vessels, and nerves. The incidence of R2 resection in primary RPS is estimated to be approximately 5% [44]. Similarly, the effect of treatment in a non-reference center on the risk of R2 margins was demonstrated, as was the impact of surgical technique on margin outcomes [45]. A study of intraoperative causes of gross resection found that 3.7% of patients had R2 resections, with liposarcoma being the most common subtype (69%). The leading causes of R2 resections were vascular involvement, unresectable peritoneal metastases, and invasion of critical organs [46]. However, it should be noted that in this study, 38% of the patients received neoadjuvant chemotherapy, which may suggest a high local progression of the tumour at baseline and could have influenced these results [46]. Furthermore, another study found that R2 resection was present in 3.28% of patients who underwent surgery for RPS. The study also revealed that R2 resections were more likely to be associated with higher grade sarcoma (OR 1.8; 95%CI 1.1–3.2, P = 0.03) and a size 20 cm (OR 2.1; 95%CI 1.1–4.2, P = 0.03) [47].

## Management of R2 resection

### Reoperation immediately after the R2 operation

Current guidelines from the National Comprehensive Cancer Network (NCCN) and ESMO clearly state that if the histopathological report shows a positive margin after STS resection of the extremity or trunk wall, re-excision with a radical R0 margin should be pursued [6, 48]. The impact of surgical margins on prognosis generally divides patients into two groups: those who have undergone resection R0 / R1 and those who have undergone resection R2, which has a much worse prognosis. It is imperative to consider further treatment as mandatory, as leaving the residual disease is a clinical mistake [49]. The opinion of experts from various reference centres is also unanimous on this issue, stating that re-excision is mandatory and additional perioperative radiation therapy or chemotherapy should be used. If radical resection is impossible, sparing resection with additional perioperative procedures is recommended to reduce the risk of recurrence and dissemination [8•]. Similar guidelines have been used for PPR, but when radical resection is not possible, additional therapy is recommended, and for patients with stage IA, close observation is an option [48]. Additionally, procedures performed in high reference compared to low reference centres are recommended in the case of high-grade sarcomas, as an improvement in 10-year OS has been proven among this group of patients (p=0.03) [50]. Re-excision improves surgical margins in 69% of patients [15]. Adequate resection in cases of gross tumour presence can increase local control (LC) among patients with STS. Research has shown that patients who underwent re-excision had local control rates (LCR) of 85%, 85%, and 82% at 5, 10, and 15 years, respectively. On the contrary, patients who did not undergo resection had LCRs of 78%, 73% and 73% (P = 0.03), respectively [51]. Numerous scientific studies have confirmed the benefit of re-excision regarding both RFS and OS [36]. In patients with synovial sarcoma with re-excision after inadequate resection, significantly better RFS was achieved than in those without re-excision (P < 0.001). A statistically significant impact of re-excision on metastatic-free survival (MFS) was also demonstrated [52]. A significant benefit of re-excision was observed for 5-year RFS and OS, with patients who underwent re-excision achieving an RFS of 87.90% compared to 49.9% for those who did not (p=0.002; RR 4.4, 95% CI 1.7-11.2), and for OS 92.10% vs. 78.30% (p=0.02; RR 4.7, 95% CI 1.2-17.8) [53]. The NETSARC analysis confirmed these results as R2 re-operated patients had superior LRFS and OS (p=0.01); no significant improvement in MFS was seen [54]. NETSARC analysis also confirmed an OS benefit in patients who underwent re-excision compared to those who did not (p<0.0001; HR 0.36, 95%CI 0.23-0.56) [55]. A meta-analysis illustrating the impact of inadequate STS resections showed that patients with residual tumour had significantly worse LRFS (OR = 3.36; 95% CI 1.97-6.44; p < 0.001), higher risk of developing distant metastasis (OR = 3.42; 95% CI 2.54-5.01; p < 0.001) and lower OS (OR = 2.26; 95% CI 1.63-3.14; p < 0.001). Re-excision equalized these results compared to elective resection in terms of LRFS and showed favorable results in the case of MFS (OR = 0.56; 95% CI 0.46-0.69; p < 0.001) [56]. Even in cases of small tumours with a diameter of less than 5 cm, immediate resection significantly affects the prognosis. A group of 59 patients after R2 resection showed that the 5-year MFS rate was 89.1% in patients with additional excision and 39.2% in those without it. The impact was also significant in the case of LCR, since local recurrence-free survival was worse (52.6%) in patients without additional excision than in those with additional excision (92.8%) [57].

The optimal time to re-excision remains to be determined. The average time to the start of treatment (TTI) is 30 days [58] and varies according to tumour grade and between STS and bone tumours [59]. Recently, there has also been a relative increase in TTI [60]. However, these results should be interpreted with reservations due to differences in reporting when institutions provide TTI 0 days immediately after surgery without taking into account previous diagnostics [61]. As stated previously, in some cases, radical resection may not be possible due to tumour expansion. In such cases, an R2 debulking procedure may be necessary [46]. Although when possible re-excision is mandatory in the case of R2 resection, there have been multiple reports on possible side effects, impact on the patient's mobility, increased risk of amputation and mutilation, and the need for plastic surgery [62–64].

### Radiotherapy after R2 operation

Presently, radiation therapy is recommended for patients with an increased risk of LR of resected STS, especially if positive margins are expected or have occurred after surgery. However, if R2 resection is anticipated due to the involvement of crucial structures, preoperative radiotherapy is superior to postoperative RT [65]. Positive margins after resection are one of the most important factors determining the use and method of radiation therapy in STS [65, 66]. In patients with positive margins treated with RT, higher doses of 6400 cGy to 6800 cGy have been associated with better local tumour control compared to doses of <6000 cGy [67]. Some histological subtypes of STS require individualized treatment, e.g., myxofibrosarcoma with an infiltrative growth pattern, which requires adjuvant radiation therapy to achieve local control [68]. Well-differentiated liposarcoma, on the other hand, does not require additional radiotherapy even after surgery with a positive margin, due to its benign nature [69]. Numerous studies have shown the beneficial effect of additional radiation therapy in reducing local recurrence rates and OS after nonradical resection [70, 71]. 5-year LC rates were shown to be significantly higher in the group with vs. without additional radiation therapy (74% vs. 56%; p=0.01), but this effect was not sustained in the case of OS and RFS [72]. LR was lower in patients who received postoperative radiotherapy compared to those who did not (p=0.0028), again without effect on OS [73]. A long-term benefit expressed as a better 10-year LRFS was also observed in patients treated with radiotherapy after inadequate R2 resection (61.3% vs 45.8%) [74]. In this group, radiotherapy showed the most significant effectiveness after inadequate resection at a dose greater than 64 Gy. The rates for 5-year LC, disease-free survival (DFS) and OS were 76%, 46.7%, and 65.2%, respectively [66]. Additionally, a postoperative boost of 1600 cGy resulted in a 5-year LRFS of 90.4% compared to patients who received preoperative radiation therapy with only an LRSF of 73.8% (p=0.13) [75]. However, even with the standard dose of 5000 cGy, positive long-term results were achieved with a 5-year local control, RFS and OS of 95% (95% CI, 80-99), 86% (95% CI, 69-94) and 94% (95% CI, 79-99), respectively [76]. In general, postoperative boost in nonradical resections does not significantly affect local control when preoperative radiotherapy is used [77]. Some studies even suggest that adequate local control and a reduction in the risk of metastases are only achieved when radiation therapy is used only as an adjunct to nonradical surgery [78]. A study examining the relationship between surgical margin width, LR, and radiotherapy showed no benefit in patients with positive margins, but for margins <1 mm, LR was 5.7% without radiotherapy and 0% with preoperative radiotherapy [79]. In the case of entirely unplanned surgery (whoops operation), the addition of radiation therapy also significantly reduces the likelihood of LR: patients who received radiation therapy before reoperation had LR in 14.3% of cases, while patients who underwent reoperation before RT had LR in 56.25% (p = 0.001) [80•]. Using postoperative radiotherapy, regardless of resection status in RPS, resulted in a significantly reduced LR rate. 5-year LRFS rates were 25.8% for radiation and 75.7% for non-radiation. There was also no effect on 5-year OS [81].

The optimal timing of radiotherapy with resection after the R2 procedure is debatable. Specifically, it is unclear whether the procedure should be performed as soon as possible or whether it can be initiated with radiation. An analysis was conducted to compare preoperative and postoperative RTH in these cases. The results did not show significant differences in LCR between the two groups. There were no statistically significant differences in LC, metastatic control, DFS, or disease-specific survival ratios. However, the preoperative dose was lower (50 vs. 60 Gy), which may have resulted in a slight trend of fewer complications in this group of patients [82].

Radiation therapy can also be the only treatment after R2 resection for residual unresectable tumours. It is also a viable option for older patients with multiple comorbidities who cannot undergo extensive re-excision. A recent systematic review evaluated the outcomes of patients with STS resected with unresectable or R2 who underwent definitive radiotherapy. The conclusion was that long-term disease control could be achieved in a subset of patients, with 5-year PFS and OS at 0-39% and 24.7-63%, respectively. The review also found that a dose of 64-66 Gy appeared to be sufficient in this indication, and doses higher than or equal to 68 Gy resulted in serious complications of therapy [83]. The definitive radiotherapy analysis included 16 patients after R2 resection. Most of these patients had unresectable disease, were not candidates for surgery, or did not consent to amputation. Most patients achieved SD; the ORR was 22% of the entire cohort. Therapy benefit was demonstrated, and the OS of the R2 group was 34.5 (95% CI 13.0-Not reached). No significant therapy-related complications were observed, and the chemotherapy used did not significantly affect the treatment outcomes of patients [84].

### Chemotherapy after R2 operation

The use of postoperative chemotherapy in treating localized STS is controversial. Conflicting conclusions from several studies have resulted in varied practice patterns in sarcoma reference centres. Furthermore, there is a lack of studies assessing cohorts of patients based on the status of surgical margins. As a result, there are no established guidelines for the use of chemotherapy after R2 resection, both in the limb and trunk and in the retroperitoneal STS. There is also insufficient data available to determine the impact of postoperative chemotherapy on the prognosis of this group of patients. However, patients after R2 resection tend to receive postoperative chemotherapy more frequently [47]. Several clinical trials have attempted to establish the role of perioperative chemotherapy in STS. The pooled analysis of the clinical trials of EORTC 62771 and EORTC 62931 established an OS benefit with adjuvant chemotherapy (HR 0.64) for patients with non-radical microscopically resection (R1), which may imply similar results for the group of patients after resection R2 [85]. Further analysis in the subgroup of patients with R2 resection is necessary as the results from clinical trials did not stratify patients according to the radicality of resection. The effect of adjuvant chemotherapy on the outcome of patients with high-grade STS of the extremities was evaluated in a group of 80 patients, including 15 who underwent nonradical tumour resection. Compared to radiotherapy alone, chemoradiation based on the MAID regimen (doxorubicin, ifosfamide, dacarbazin, and mesna) or doxorubicin alone showed a benefit in LRFS (83.3% vs 33.3% for adjuvant chemoradiation and adjuvant radiotherapy, respectively; p = 0.06). No effect was seen on distant recurrence-free survival (p = 0.22), disease-free survival (p = 0.63) or overall survival (p = 0.53). In particular, none of the patients in the chemoradiation group experienced local recurrence [86]. A panel of experts convened to create recommendations in controversial cases related to STS. A consensus was reached regarding managing nonradical resections with macroscopically residual tumours. For extremity and trunk wall sarcomas, experts agreed on the need for additional therapy, primarily radiation therapy. Furthermore, 32% supported the use of chemotherapy after nonradical surgery. Regarding PPR, more than half of the experts supported chemotherapy [8•]. These results are consistent with the results in patients with RPS after R2 surgery, where the benefit of chemotherapy was shown only in 3-year OS, with a deterioration in long-term survival (p=0.024; HR 0.69, 95% CI 0.50-0.95 vs. p=0.009; HR 2.15, 95% CI 1.21-3.81) [47]. Therefore, the use of chemotherapy in the adjuvant setting for R2 resection should be used with caution. It is important to consider the individual clinical situation of the patient and the risk of distant metastases.

## Summary

**Fig. 3:**
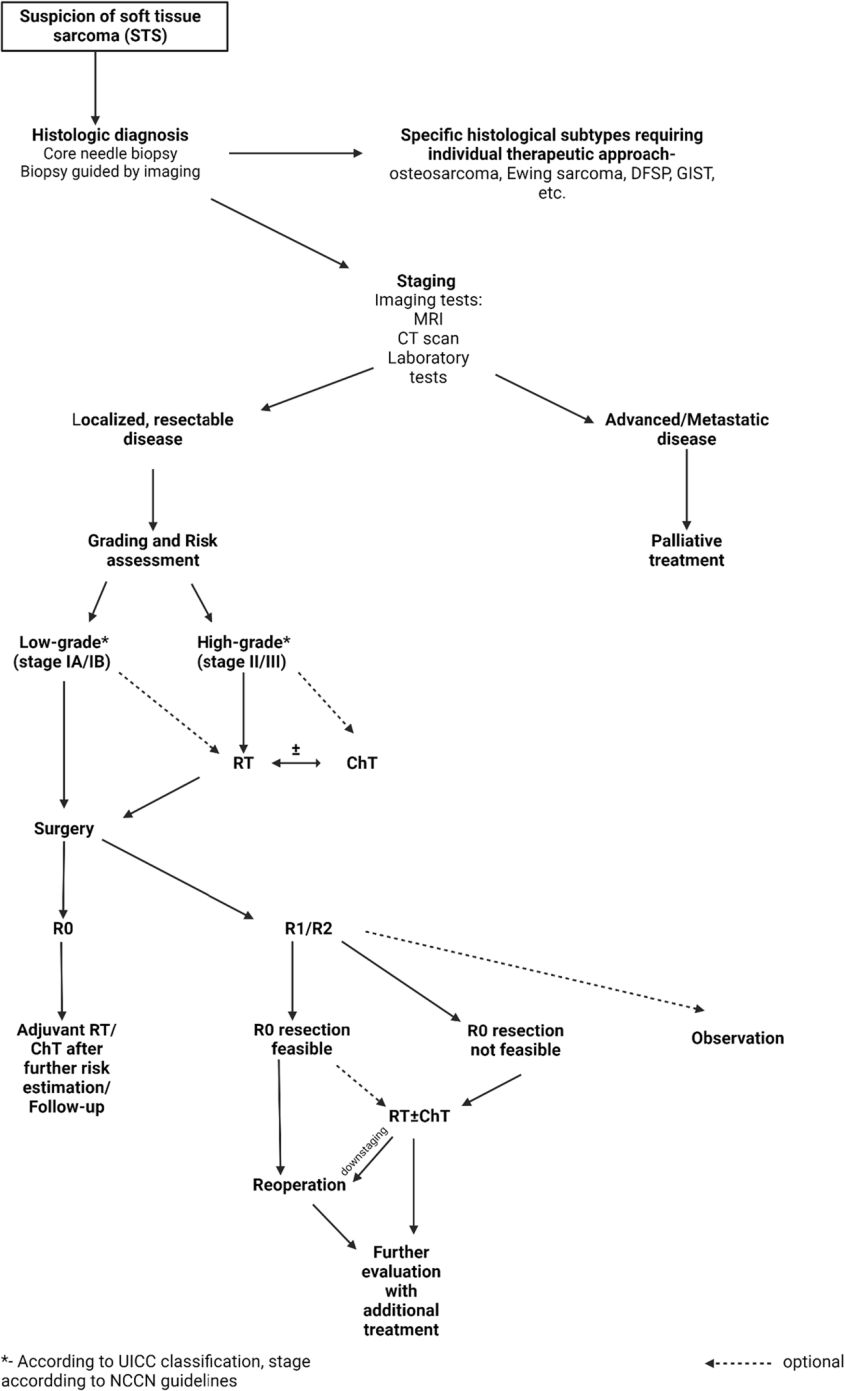
Algorithm for non-radical resection in STS of extremity and trunk wall. RTH-radiotherapy; ChT-chemotherapy.

In the last decades, the multi-modality approach has improved the outcomes of STS treatment. Surgery remains the cornerstone of curative treatment. Many factors can influence surgical outcomes, leading to nonradical resections. The management of this scenario remains to be established. Currently, the treatment of choice is reoperation with possible additional radiation / chemotherapy (Figure 3). This significantly reduces the risk of local recurrence, but survival data are incomplete and sometimes conflicting. In some less aggressive, low-grade subtypes, only careful observation of patients is possible. Radiotherapy prior to resection has clear advantages over local control and should be offered to most patients. A potential relatively new solution is the use of radiation therapy as the sole adjuvant treatment after R2 resection, but this option requires more direct comparisons in an appropriately selected group of patients. However, chemotherapy has no proven role in this setting and its use seems justified only in high-grade, high-risk tumours. The management strategy should be individualized, but resection margins are one of the main risk factors for recurrence and poor survival, so it is advisable to assess the risk in patients after nonradical resection and manage it by re-excision and possibly radiotherapy.

## Data Availability

No datasets were generated or analysed during the current study
